# Using CT colonography as a triage technique after a positive faecal occult blood test in colorectal cancer screening

**DOI:** 10.1136/gut.2009.176867

**Published:** 2009-08-04

**Authors:** M H Liedenbaum, A F van Rijn, A H de Vries, H M Dekker, M Thomeer, C J van Marrewijk, L Hol, M G W Dijkgraaf, P Fockens, P M M Bossuyt, E Dekker, J Stoker

**Affiliations:** 1 Academic Medical Centre Amsterdam, The Netherlands; 2 Radboud University Nijmegen Medical Centre, The Netherlands; 3 Erasmus Medical Centre Rotterdam, The Netherlands

## Abstract

**Objective:**

The purpose of this study was to evaluate the effectiveness of CT colonography (CTC) as a triage technique in faecal occult blood test (FOBT)-positive screening participants.

**Methods:**

Consecutive guaiac (G-FOBT) and immunochemical (I-FOBT) FOBT-positive patients scheduled for colonoscopy underwent CTC with iodine tagging bowel preparation. Each CTC was read independently by two experienced observers. Per patient sensitivity, specificity and positive and negative predictive values (PPV and NPV) were calculated based on double reading with different CTC cut-off lesion sizes using segmental unblinded colonoscopy as the reference standard. The acceptability of the technique to patients was evaluated with questionnaires.

**Results:**

302 FOBT-positive patients were included (54 G-FOBT and 248 I-FOBT). 22 FOBT-positive patients (7%) had a colorectal carcinoma and 211 (70%) had a lesion ⩾6 mm. Participants considered colonoscopy more burdensome than CTC (p<0.05). Using a 6 mm CTC size cut-off, per patient sensitivity for CTC was 91% (95% CI 85% to 91%) and specificity was 69% (95% CI 60% to 89%) for the detection of colonoscopy lesions ⩾6 mm. The PPV of CTC was 87% (95% CI 80% to 93%) and NPV 77% (95% CI 69% to 85%). Using CTC as a triage technique in 100 FOBT-positive patients would mean that colonoscopy could be prevented in 28 patients while missing ⩾10 mm lesions in 2 patients.

**Conclusion:**

CTC with limited bowel preparation has reasonable predictive values in an FOBT-positive population and a higher acceptability to patients than colonoscopy. However, due to the high prevalence of clinically relevant lesions in FOBT-positive patients, CTC is unlikely to be an efficient triage technique in a first round FOBT population screening programme.

Colorectal carcinoma (CRC) is the second leading cause of cancer deaths in the USA and many other countries, with an approximate lifetime risk of 6%.^1^ Most CRCs are assumed to develop from benign, neoplastic adenomatous polyps.^2^ Early detection and treatment of CRCs and colorectal adenomas could reduce mortality. Therefore, several countries are currently investigating or have already started a CRC screening programme using a faecal occult blood test (FOBT). The FOBT is a cost-effective, safe test that is acceptable to patients and that detects more cancers at a less advanced stage than would have presented symptomatically.^3^ Screening with FOBT has been demonstrated to reduce CRC-related mortality by 14–16% over 10–18 years.^4^,^5^,^6^

Similar to other screening tests, such as mammography or PAP smear, the FOBT generates a considerable number of false positives. In CRC screening trials, between 0.8% and 15% of participants tested had a positive FOBT result, while 55–65% of participants with a positive FOBT result had no CRC or adenoma.^3^,^7^,^8^ As a result these participants undergo an unnecessary colonoscopy, which is considered by many individuals as an investigation with significant burden and risk of complications.^9^ A potential solution to reduce this number of unnecessary colonoscopies would be the introduction of a triage instrument. A prerequisite for using a triage instrument is that it has the ability to identify correctly participants without CRC or large polyps in those with an FOBT-positive result. With a very high negative predictive value (NPV), the number of FOBT-positive patients receiving a colonoscopy could be reduced while no cases with CRC or large polyps would be missed. CT colonography (CTC) has been shown to have good per patient test characteristics in detecting CRC and large polyps.^10^,^11^,^12^ Its per patient sensitivity was 96% in the detection of colorectal cancer, with a sensitivity of 93% in identifying polyps ⩾10 mm and 86% for polyps ⩾6 mm. The specificity for polyps ⩾10 mm was 97% and for polyps ⩾6 mm 86%.^10^

Good adherence to a population screening examination can be obtained if the offered screening method is highly acceptable to patients.^13^ Previous studies have shown that CTC examinations are experienced as less burdensome than colonoscopy.^9^,^14^ The burden of the CTC may be reduced even further if the examination is performed without an extensive bowel preparation as is required for colonoscopy.^15^,^16^,^17^

So far, the accuracy and acceptability to patients of CTC have only been evaluated in a screening setting and in a high-risk population, not in FOBT screening-positive patients as a triage technique. In this study we evaluated the use of CTC in an FOBT-positive screening population in terms of its diagnostic accuracy, positive predictive value (PPV) and NPV, and its burden, relative to colonoscopy.

## Methods

### Study population

In two FOBT screening pilot studies in The Netherlands a cohort of approximately 30 000 individuals between 50 and 75 years of age received an FOBT test at home, of which half received a non-rehydrated guaiac test (G-FOBT; Haemoccult II, Beckman Coulter, Fullerton, California, USA) and the other half received a semi-quantative immunochemical test with a cut-off level of 50 ng/ml for positive testing (I-FOBT, OC-sensor, Eiken Chemical, Tokyo, Japan). This was the first pilot study of CRC screening in The Netherlands, thus invitees had not received any other CRC screening test previously. The results of this FOBT pilot study have been reported in detail elsewhere.^18^,^19^ The FOBT-positive patients scheduled to undergo colonoscopy were invited to undergo a CTC before the colonoscopy at the Academic Medical Centre of Amsterdam, Radboud University Nijmegen Medical Centre or the Erasmus Medical Centre of Rotterdam, The Netherlands. Exclusion criteria were: terminal illness, severe psychiatric symptoms, colonoscopy or another FOBT in the previous 2 years, examinations for research purposes with radiation exposure in the last 12 months, iodine contrast allergy, hyperthyroidism and pregnancy. The CTC study had been approved by the institutional review boards of the three institutions and written informed consent was obtained from all participants.

### CT colonography

#### Bowel preparation

A non-cathartic bowel preparation was used to reduce patient discomfort. Two different bowel regimes were used. The first 153 participants received preparation 1 and the following 149 participants received preparation 2. Preparation 1 started 2 days before CTC and consisted of ingestion of 50 ml of high-osmolar ionic monomer meglumine ioxithalamate (Telebrix Gastro 300 mg I/ml; Guerbet, Cedex, France) with each meal ending with 50 ml 1.5 h before CTC (total 350 ml). In addition, patients followed a low-fibre diet for 2 days and took only liquids on the evening and morning before CTC. Preparation 2 started 1 day before CTC with the low-fibre diet and 50 ml of Telebrix four times (total 200 ml). The amount of ingested contrast agent was reduced during the second half of this study because new publications on CTC bowel preparation showed that only 1 day of bowel preparation results in good image quality and polyp detection.^15^,^16^,^20^

#### CTC technique

Examinations were performed using a low dose protocol with 40 or 32 reference mAs on two 64-slice CT scanners (table 1). Participants were scanned in the supine and prone position. A muscle relaxant, 20 mg of butylscopolamine bromide (Buscopan; Boehringer-Ingelheim, Ingelheim, Germany) or, when contraindicated, 1 mg of glucagon hydrochloride (Glucagen; Novo-Nordisk, Bagsvaerd, Denmark), was injected immediately prior to insufflation of the colon. A flexible balloon-tipped rectal catheter (20 French gauge) was inserted to insufflate approximately 3 litres of CO_2_ gas into the colon, using an automated insufflator (ProtoCO_2_l, Bracco Diagnostics, Princeton, New York, USA). No intravenous contrast medium was administered.

**Table 1:** CT parameters

	Philips Brilliance*	Siemens SOMATOM Sensation†
Collimation	64×0.625 mm	64×0.6 mm
Tube voltage	120 kV	120 kV
Pitch	1.2	1.4
Reference mAs	40 mAs	32 mAs
Slice thickness	0.9 mm	1.0 mm
Rotation time	0.4 s	0.5 s
Dose modulation	*z*-axis	CARE Dose 4D‡

*Brilliance, Philips Medical Systems, Best, The Netherlands.

†SOMATOM Sensation, Siemens Medical Solutions, Munich, Germany.

‡CARE dose 4D incorporates *x*–*y* and *z*-axis modulation.

#### CTC image analysis

Because of the restricted bowel regime and the presence of tagged stool, a primary 2D axial evaluation (primary window setting 1500, −250 HU) was carried out with 3D problem solving for the detection of polyps. This was performed on a workstation with specialised software (View Forum, Philips Medical Systems, The Netherlands; Aquarius Workstation, TeraRecon, San Matteo, California, USA). Two of seven experienced observers (radiologists and research fellows) who had each evaluated at least 100 CTCs verified by colonoscopy (range 100–700 CTCs) identified lesions in the FOBT-positive participants. The results of two observers were combined: CTC was considered positive if at least one observer had detected a lesion (“double reading”). This approach was used to enhance detection as CTC is used as a triage technique for which sensitivity and NPV are critical. The chance of missing a relevant lesion in an FOBT positive should be minimised.

Lesions were measured at the multiplanar reformatted (MPR) setting that showed the maximal diameter of the detected lesion. For each lesion, the location, morphology, size and probability (on a 5-point scale: 0, 25, 50, 75 or 100%) were annotated. Flat polyps were defined as lesions that protrude <2.5 mm from the adjacent mucosa.^21^ Only lesions ⩾6 mm that the observer reported with a ⩾50% probability were considered positive and unblinded at colonoscopy. Quality of bowel preparation was rated on a scale from 1 (uninterpretable images due to untagged faeces) to 5 (excellent preparation with almost no untagged faeces) by each observer. When the CTC was judged insufficient for evaluation by two observers, the patient was excluded for analysis. All CTCs were also interpreted on extracolonic findings by one of five gastrointestinal radiologists. Findings were classified according to the CTC Reporting and Data System (C-RADS; for classification of categories see table 5).^22^

**Table 5:** Extracolonic findings in FOBT-positive participants

C-RADS classification*	No of participants†	Type of E4 findings‡	Additional procedures
E1	138 (42.6%)	–	
E2	164 (50.6%)	–	
E3	13 (4.0%)	–	Imaging: 3
E4	10 (3.1%)	Aortic aneurysm: 2	Imaging: 12
		Iliac aneurysm: 1	Operation: 2
		Extracolonic mass: 8	
		Lung nodules: 2	

*C-RADS classification:^22^ E1 normal exam or anatomical variant; E2 clinically unimportant finding (eg, liver or kidney cysts); E3 probably unimportant finding (eg, indeterminate renal lesions); E4 potentially important finding (eg, aortic aneurysm, solid mass in liver or kidney).

†Numbers represent all participants that received a CTC scan (thus also participants that refused a colonoscopy after CTC and participants with a CTC that was of insufficient quality for polyp detection)

‡All extracolonic findings found in 9 participants.

C-RADS, CTC Reporting and Data System; FOBT, faecal occult blood test.

### Colonoscopy

Within approximately 2 weeks (mean 11 days; SD 10 days) after CTC, a colonoscopy was performed. Bowel preparation consisted of 4 litres of polyethylene glycol electrolyte solution (KleanPrep; Helsinn Birex Pharmaceuticals, Dublin, Ireland) or 4 litres of macrogol solution (Colofort; Laboratoires Macors, Auxerre, France) and a clear liquid diet starting the evening before colonoscopy. Experienced gastroenterologists, gastroenterology fellows and colonoscopy nurses with supervision performed optical colonoscopy with a standard colonoscope (Olympus, Tokyo, Japan). Sedation (midazolam, Dormicum; Roche, Basel, Switzerland), analgesics (fentanyl, Fentanyl-Janssen; Janssen Pharmaceuticals, Beerse, Belgium) and a muscle relaxant (butylscopolamine bromide, Buscopan; Boehringer-Ingelheim, Ingelheim, Germany) were commonly used in all patients (only 2% refused sedation).

During the withdrawal of the colonoscope starting from the caecum, the colonoscopy was videotaped and the findings of the CTC were revealed to the colonoscopist after completing the examination of one colonic segment. This technique is called “segmental unblinding” and leads to an enhanced reference standard due to combination of CTC and colonoscopy results. Polyp size was estimated by an opened biopsy forceps or by a linear measure probe (Olympus, Tokyo, Japan). In participants with an incomplete colonoscopy, the colonoscopy was repeated at a later time point. The histology of the lesion biopsies was classified as normal, hyperplastic, adenoma (serrated, tubular, tubulovillous or villous) or carcinoma according to the Vienna classification.^23^ Advanced adenomas were defined as adenomas ⩾10 mm, with high-grade dysplasia or with a villous component >20%.^24^

### Questionnaires and participation

Six standardised questionnaires, also used in previous CTC studies,^9^,^25^ were given to all participants. Questionnaire 1 was to be completed at home before both examinations, questionnaires 2–5 before and just after CTC and colonoscopy, and questionnaire 6 was sent 5 weeks after colonoscopy. In questionnaire 1, participants were asked about their demographic characteristics. In questionnaire 2 (before the CTC) and questionnaire 4 (before colonoscopy) questions about discomfort of bowel preparation were asked on a 5-point scale (1 = no burden, 2 = mild, 3 = moderate, 4 = severe or 5 = extreme burden). Questions about discomfort of the examination were also asked after the examinations on a 5-point scale (questionnaire 3 and 5, respectively). In questionnaires 5 and 6, participants were asked which examination or bowel preparation they found most burdensome and what examination they would prefer in the future (answered on a 7-point scale: 1 = definitely CTC to 7 = definitely colonoscopy).

The participation rate was calculated for all FOBT-positive patients who attended the outpatient clinic. Reasons for not participating in the CTC study were noted for all FOBT-positive subjects who attended the outpatient clinic.

### Statistical analysis

The sensitivity, specificity, PPV and NPV of CTC using a CTC lesion size cut-off of ⩾10 mm and ⩾6 mm were calculated on a per patient basis for two size categories: lesions ⩾10 mm and lesions ⩾6 mm found at colonoscopy. This was done for both G-FOBT and I-FOBT at a 50 and 100 ng/ml cut-off. For our calculations the largest polyp size measured by CTC by the two observers was used when calculating the accuracy of CTC as a triage technique. A patient with a matched polyp that measured 4 mm at CTC, for example, but was measured as 6 mm at colonoscopy was considered as a false negative (using a ⩾6 mm cut-off at CTC). Furthermore, we calculated the PPV and NPV for different CTC cut-off values and plotted them on a graph. Per polyp sensitivity for colonoscopy was calculated using the false-negative lesions at colonoscopy (known after unblinding of the CTC results). Answers on acceptability of the technique to patients and degree of burden between CTC and colonoscopy were compared using ordinal regression analysis. Differences in quality of bowel preparation were tested with the χ^2^ test.

## Results

**Figure 1:**
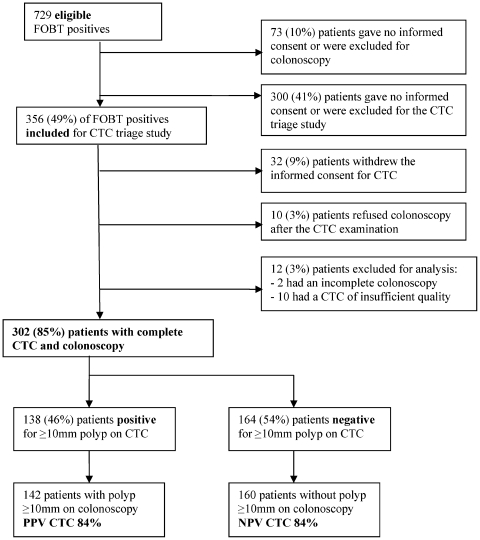
Flowchart of faecal occult blood test (FOBT)-positive participants. CTC, CT colonography; NPV, negative predictive value; PPV, positive predictive value.

Between June 2006 and May 2008, 356 participants were included in the CTC triage study in the participating centres. In fig 1 a flowchart is given, presenting the numbers of participants that gave informed consent and the numbers of excluded participants. The data of 302 participants were complete for analysis. The mean age of the participants was 61 years (SD 6). Further demographic characteristics of the participants are given in table 2. A total of 248 participants had a positive I-FOBT and 54 participants had a positive G-FOBT. This difference was due to the differences in FOBT participation rate (I-FOBT had a 12.7% higher participation rate) and FOBT positivity rate; 2.4% of returned tests was positive for the G-FOBT and 8.5% for the I-FOBT with 50 ng/ml cut-off.^18^,^19^,^26^

**Table 2:** Demographic characteristics and FOBT type

Mean age in years (SD)	61 (6)
Male/female (ratio)	187/115 (1.6:1)
Ethnicity: total number of whites	291 (97%)
Highest education level:	
Primary school	20 (7%)
High school	27 (9%)
Vocational education	173 (57%)
University	77 (25%)
Not provided	5 (2%)
Nett income per month	
<US$2059/>US$2059/not provided	88/131/83
FOBT:	
G-FOBT	54 (18%)
I-FOBT	248 (82%)

FOBT, faecal occult blood test; G-FOBT, guaiac FOBT; I-FOBT, immunochemical FOBT.

### FOBT and colonoscopy results

CRC was found in 22 participants (7%); these were 14 participants in the I-FOBT group (PPV CRC 6%) and 8 participants in the G-FOBT group (PPV CRC 15%). A total of 208 lesions ⩾10 mm were found in 142 participants (47%) and 398 lesions ⩾6 mm in 211 participants (70%). The PPV for lesions ⩾10 mm in the G-FOBT-positive group was 59% vs 44% in the I-FOBT-positive group. For lesions ⩾6 mm the PPVs were 67 and 70% respectively. In table 3 the distribution of lesions per histology type is given (see van Rossum *et al*^18^ for more details on the FOBT results). In total, 14 lesions ⩾6 mm were found at colonoscopy after unblinding of the CTC results, and thus were false negative for colonoscopy. This results in a per polyp sensitivity for colonoscopy of 96% for lesions ⩾6 mm. In 11 participants (3.6%) bleeding followed after polypectomy during colonoscopy for which one participant needed a hospital stay of one night. In none of the participants did a perforation occur.

**Table 3:** Information on histology types of all removed lesions at colonoscopy

	All FOBT-positive subjects	I-FOBT 50 ng/ml (248 participants)	G-FOBT (54 participants)
Carcinoma	22	14	8
Adenoma	574	473	101
Hyperplastic polyp	207	182	25
Hamartoma	1	1	0
Inflammatory polyp	4	4	0
Lipoma	3	3	0

FOBT, faecal occult blood test; G-FOBT, guaiac FOBT; I-FOBT, immunochemical FOBT.

### CT colonography

There were no complications at CTC. The CTCs of 10 participants were rated of insufficient quality for evaluation. The quality of bowel preparation in both preparation groups was not rated significantly different.

#### Sensitivity and specificity

When using a CTC cut-off ⩾10 mm, the per patient sensitivity of CTC was 82% (95% CI 74% to 89%) and the specificity was 86% (95% CI 80% to 93%) for finding lesions at colonoscopy ⩾10 mm. One participant with a carcinoma was missed at CTC (sensitivity 95%) and 24 participants (17%) with an adenoma of ⩾10 mm were missed. Twenty-three of these adenomas measured between 10 and 16 mm at colonoscopy and one measured 30 mm. The missed carcinoma was a flat rectal carcinoma that was even retrospectively not visible at CTC. In the 24 participants with a missed adenoma, 20 of these had a lesion that was detected at CTC but measured between 6 and 9 mm, thus being smaller than the 10 mm cut-off. In table 4 results for sensitivity and specificity are shown for I-FOBT with 50 and 100 ng/ml cut-off and for G-FOBT separately.

**Table 4:** Per patient sensitivity, specificity, positive and negative predictive values for CT colonography (CTC) per lesion size category

	Both FOBT		I-FOBT 50 ng/ml		I-FOBT 100 ng/ml		G-FOBT	
	%	Ratio (95% CI)	%	Ratio (95% CI)	%	Ratio (95% CI)	%	Ratio (95% CI)
Lesions ⩾10 mm*								
Sensitivity	82	116/142 (74 to 89)	80	88/110 (72 to 88)	81	69/85 (74 to 89)	88	28/32 (81 to 94)
Specificity	86	138/160 (80 to 93)	86	119/138 (79 to 93)	88	58/66 (81 to 94)	86	19/22 (80 to 93)
PPV	84	116/138 (77 to 91)	82	88/107 (75 to 90)	90	69/77 (84 to 96)	90	28/31 (85 to 96)
NPV	84	138/164 (77 to 91)	84	119/141 (77 to 92)	78	58/74 (70 to 86)	83	19/23 (75 to 90)
Lesions ⩾6 mm†								
Sensitivity	91	192/211 (85 to 91)	90	157/174 (84 to 96)	90	100/111 (84 to 96)	94	34/36 (90 to 99)
Specificity	69	63/91 (60 to 89)	72	53/74 (63 to 80)	68	27/40 (57 to 77)	56	10/18 (46 to 65)
PPV	87	119/220 (80 to 93)	88	157/178 (82 to 95)	88	100/113 (82 to 95)	81	34/42 (73 to 89)
NPV	77	63/82 (69 to 85)	76	53/70 (67 to 84)	71	27/38 (62 to 80)	83	10/12 (76 to 91)

*Detection of lesions ⩾10 mm at colonoscopy using a CTC size cut-off of ⩾10 mm. †Detection of lesions ⩾6 mm at colonoscopy using a CTC size cut-off of ⩾6 mm.

FOBT, faecal occult blood test; G-FOBT, guaiac FOBT; I-FOBT, immunochemical FOBT; NPV, negative predictive value; PPV, positive predictive value.

When using a CTC cut-off ⩾6 mm the per patient sensitivity of CTC was 91% (95% CI 85% to 97%) and the specificity was 69% (95% CI 60% to 78%) for finding lesions at colonoscopy ⩾6 mm.

Again the participant with the flat rectal carcinoma was missed using this cut-off; 15 participants (8%) with an adenoma of ⩾6 mm and 6 (5%) with an adenoma of ⩾10 mm were missed.

#### Positive and negative predictive values

The PPV of CTC was 84% (95% CI 77% to 91%) for the detection of lesions ⩾10 mm found at colonoscopy, when using a cut-off ⩾10 mm at CTC. The NPV using this cut-off was 84% (95% CI 77% to 91%). Using a cut-off of ⩾6 mm (for CTC and colonoscopy lesions) the PPV of CTC was 87% (95% CI 80% to 93%). An NPV of 77% (95% CI 69% to 85%) corresponded to this cut-off. Using CTC triage with a 10 mm cut-off in 100 FOBT-positive patients would mean that colonoscopy could be prevented in 54 patients, while missing ⩾10 mm lesions in 9 patients. For a 6 mm cut-off this would mean prevention of colonoscopy in 28 patients, while missing ⩾10 mm lesions in 2 patients.

**Figure 2:**
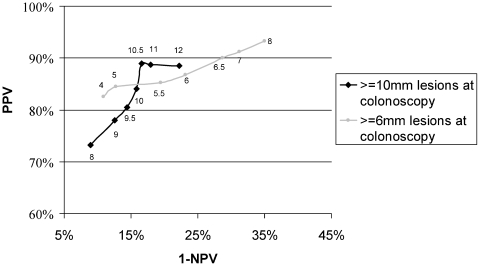
Plot of the positive predictive value (PPV) versus the negative predictive value (NPV) when using different cut-off sizes for CT colonography (CTC) for detection of true colonoscopy lesions of ⩾10 mm and ⩾6 mm. The curve shows a plot of PPV versus 1–NPV. Results for detection of patients with lesions on colonoscopy of ⩾10 mm, for cut-off sizes for CTC lesions of ⩾8, 9, 9.5, 10, 10.5, 11 and 12 mm are shown. Results for detection of lesions of ⩾6 mm are shown for CTC cut-off sizes of 4, 5, 5.5, 6, 6.5, 7 and 8 mm.

Figure 2 shows a comparison of PPV and NPV for different CTC cut-off values. A cut-off value of 9.5 mm for CTC had a PPV of 81% and an NPV of 86% for the detection of colonoscopy lesions ⩾10 mm; for 10.5 mm this was 89% and 83%. For a 5.5 mm cut-off this was 85% and 81% and for 6.5 mm this was 90% and 71%, respectively, for detection of colonoscopy lesions ⩾6 mm.

#### Extracolonic findings

In total 12 E4 extracolonic findings were reported in 9 participants (2.7%). These findings had not previously been diagnosed in these patients. See table 5 for the detected extracolonic findings classified according to the C-RADS classification and the additional procedures that have been performed.

### Questionnaires and participation

**Figure 3:**
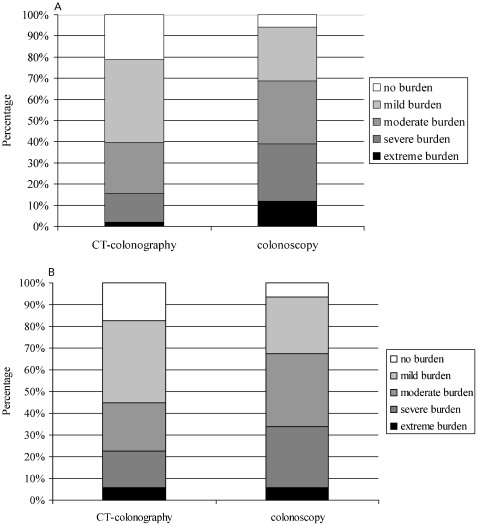
(A) Degree of burden for both examinations overall. Participants found the colonoscopy examination significantly more burdensome than the colonoscopy preparation. (B) Degree of burden from CT colonography and colonoscopy bowel preparations. No significant difference was found between the degree of burden from the colonoscopy bowel preparation and the CT colonography bowel preparation.

When comparing the two examinations, 16% of all participants experienced the CTC examination as extremely or severely burdensome versus 41% for the colonoscopy examination (p<0.05; see fig 3A). For the bowel preparations, 23% of all participants experienced the CTC bowel preparation as extremely or severely burdensome, compared with 34% for colonoscopy (p>0.05; see fig 3B). After 5 weeks, 85% of the participants rated the colonoscopy as the most burdensome examination of the two. A majority of 67% of all participants would choose CTC as first examination after FOBT in future screening. Of all participants that were scheduled to undergo colonoscopy, 356 (54%) were also willing to undergo CTC (see fig 1). The main reason for not participating in CTC triage was that participants did not want to undergo an unnecessary additional examination (67%).

## Discussion

CTC has proven to be an accurate technique for detection of colorectal polyps and carcinomas.^10^,^12^ This study investigated the role of CTC with a limited bowel preparation as a triage technique after positive FOBT in order to reduce the number of unnecessary colonoscopies. We found a high per patient sensitivity of CTC in the FOBT-positive subjects, especially for finding lesions ⩾6 mm at colonoscopy. The sensitivity for finding lesions ⩾10 mm was somewhat lower, which may have resulted from the fact that most patients had only one lesion of ⩾10 mm but multiple lesions of ⩾6 mm, resulting in a higher probability of detecting at least one lesion ⩾6 mm in a patient. It is important to realise, however, that most polyps of 6–9 mm are serendipitous findings because they usually do not bleed and therefore are not the main target of an FOBT screening programme.

When considering the usefulness of CTC as a triage technique, one should aim for a high NPV. NPVs in this study were fair but did not approach 100%, which would be ideal in a triage setting. When the CTC cut-off level was decreased, the NPV increased as result, but the number of false positives also increased, which is not preferable. The PPVs differed somewhat for CTC performance in the FOBT groups; the lowest PPV was found for I-FOBT with 50 ng/ml cut-off. This difference most probably occurred because of a difference in lesion prevalence in the FOBT groups, which can result in a different proportion of false positives.

One of the main reasons for performing CTC triage in FOBT-positive subjects instead of a direct colonoscopy is that the acceptability has been reported to be better for CTC than for colonoscopy.^9^,^14^ In this study too the majority of participants reported a lower burden of the total examination including bowel preparation for CTC than for colonoscopy. Furthermore, 67% of the participants would prefer CTC instead of colonoscopy as first choice for future examination.

A triage technique is only useful when the number of patients that receive the colonoscopy will be substantially reduced. In this study we found that if 100 FOBT-positive subjects undergo a CTC, 46% will have to undergo a colonoscopy when using a CTC cut-off size of 10 mm. When considering costs of the initial management only, two different strategies are possible: CTC as triage and subsequent colonoscopy in CTC-positive patients or a direct colonoscopy in all FOBT-positive patients. Using a 10 mm cut-off, CTC examination costs must not exceed 54% of colonoscopy costs. However, this cost ratio does not seem applicable when the current costs for these examinations are considered. In a recent cost-effectiveness study by Regge *et al*,^27^CTC examination costs are calculated as US$665 and colonoscopy costs as US$877. Thus here the CTC costs are 76% of the colonoscopy costs. Even when using a cut-off size of 6 mm, 73% will have to undergo colonoscopy after CTC and costs of CTC must not exceed 27% of colonoscopy costs. Hence, from an economic perspective, the use of CTC as a triage technique is most probably not efficient. Its apparent inefficiency resulted primarily from the high PPV of both FOBTs; 44% in those who were I-FOBT positive and 59% in those who were G-FOBT positive. This PPV of both FOBT-positive groups was much higher than expected considering the PPV for adenomas and cancer in earlier studies.^5^,^6^,^8^,^28^ However, a lower lesion prevalence is found when using lower cut-off levels for the I-FOBT.^28^ Furthermore, a few studies have reported a decrease in lesion prevalence in successive FOBT screening rounds.^29^,^30^ The use of CTC might then become more cost-efficient. Additionally, when calculating cost-efficiency, the false-negative lesions at colonoscopy should also be considered. In our study the per polyp sensitivity of colonoscopy was high (96%) for lesions ⩾6 mm.

When considering the positivity rate of the FOBT itself, this was similar to that found in previous studies. In a review by Hewitson *et al*,^5^ the positivity rate of the G-FOBT varied from 0.8% to 5.3%, and other studies showed I-FOBT positivity rates of 4.7% and 6.9%.^8^,^28^ In this study, 2.4% of returned G-FOBTs and 8.5% of returned I-FOBTs with 50 ng/ml cut-off were positive.^18^,^19^ A higher positivity rate of the FOBT consequently results in a higher number of false positives, which would make CTC as triage more efficient. The PPV of the I-FOBT was indeed lower than that of the G-FOBT; however, still 46% of patients in the I-FOBT group had an adenoma or cancer ⩾10 mm. Therefore, CTC triage seems not to be an efficient strategy in this first round FOBT.

In contrast to previous CTC studies, we calculated the diagnostic accuracy by using the CTC lesion size as the cut-off: participants will be referred for colonoscopy based on the size of lesions measured at CTC. This method of analysing CTC as a triage technique may give a more realistic view than the method of matching CTC and colonoscopy polyps, and then using the colonoscopy lesion size as the reference size for data reporting. In this study reporting of sensitivity or specificity per lesion histology was considered irrelevant for the evaluation of triage with CTC, because the histology cannot be defined at CTC and only polyp size can be used as an indicator for referral to colonoscopy. Polyp size is an important parameter because larger polyps (⩾10 mm) have a higher chance of malignant development.^2^,^31^ A disadvantage of this method is that differences in measurement of lesions at CTC and colonoscopy are not corrected by matching. Previous studies have shown that quite large differences in measurement of CTC and colonoscopy lesions can exist.^32^,^33^ These differences can cause an increase in the number of false positives and false negatives of CTC in the setting of triage.

The graph of the predictive values for different CTC polyp size cut-offs shows an optimal cut-off for CTC (highest PPV and NPV) at 10 or 10.5 mm and 6 or 6.5 mm, respectively (see fig 2). These cut-offs might be different in other settings with another method of measurement (2D vs 3D) and different observers.^34^,^35^ In this study, a 2D measurement was performed because a primary 2D read was carried out in the tagging-only prepared CTCs. Currently, there is lack of consensus on the optimal method of measurement; some studies showed that 2D measurement was most accurate,^34^,^36^,^37^ while others recommended 3D measurement.^35^,^38^

A known advantage of CTC compared with colonoscopy is the lower complication rate. Previous studies reported perforation rates of 0.009% for CTC in screening participants^39^ and of 0.3% for screening colonoscopy.^40^,^41^ In this study no complications occurred during CTC, whereas 11 of those screened reported rectal blood loss after colonoscopy. No perforations occurred during colonoscopy. However, we must realise that colonoscopy is not only diagnostic but also incorporates treatment. Subjects with lesions at CTC will also have to undergo colonoscopy and have similar risks of complications.

At CTC, extracolonic findings should be reported for ethical reasons.^42^ In this study group the number of highly relevant findings (E4) was low (3.1%), especially when compared with other studies with high or average risk patients, where incidences of highly relevant findings have been reported ranging between 9% and 23%.^43^,^44^,^45^ The results of our study are comparable with what was found in a large CTC screening trial with asymptomatic patients.^11^ This could be due to the low radiation dose protocols in these studies, which might result in a reduced visibility and detection of E4 findings.^46^ The significant extracolonic findings will inevitably lead to increased costs due to additional examinations and treatment when CTC is used as triage technique. Pickhardt *et al* showed that costs per screened person increased by US$98.56 due to extracolonic findings in CTC screening.^47^

A potential limitation of this study is that bowel preparation with meglumine ioxithalamate was reduced from 2 days to just 1 day after inclusion of 153 participants. This was done to diminish the burden of the bowel preparation even more and because newly published literature pointed out that 1 day preparation was sufficient.^15^,^16^,^20^ No differences in image quality were seen by the CTC observers, so this change of bowel preparation regime probably did not influence outcomes. Another potential limitation is that all CTCs were scored by two observers who varied during the research period. To facilitate a quick review process, the results of both observers were combined (double reading) and no consensus reading was performed. Due to the double reading the number of false negatives decreases and the sensitivity and NPV increase, at the expense of the specificity and PPV.^48^ A double read is more costly and might not be time-efficient for screening or triage purposes in large populations. A computer-aided detection (CAD) system could be a solution to this problem, but on the other hand the additional value of CAD for experienced readers has not been proven.^49^ A third potential limitation is that selection might have occurred between participants and non-participants in this CTC study. However, when aspects of age, gender and lesion prevalence between the FOBT-positive subjects in this study and the first original FOBT pilot study are compared, no differences are observed when considering those characteristics.^18^

In conclusion, this study shows that CTC with limited bowel preparation is unlikely to be an efficient triage technique in a first round FOBT population screening programme. The patient burden of the CTC was lower than that of colonoscopy and most participants preferred CTC to colonoscopy for future examination. However, due to the high lesion prevalence in the FOBT-positive group and the relatively high miss rate of relevant lesions at CTC, CTC should not be considered as a triage technique in this specific first round FOBT population. In further FOBT screening rounds, lesion prevalence is possibly lower and in this situation CTC could be more effective.
